# Medial Branch Radiofrequency Treatment for Low-Back Pain in Cancer Patients: A Case Series

**DOI:** 10.1089/pmr.2022.0050

**Published:** 2022-11-28

**Authors:** Ye Sull Kim, Taehoon Kim, Youngkwan Lee, Yu Jin Oh, Jeongmin Oh, A Ram Doo

**Affiliations:** ^1^ Department of Anesthesiology and Pain Medicine, Medical School, Jeonbuk National University, Jeonju, South Korea.; ^2^ Research Institute of Clinical Medicine, Jeonbuk National University-Biomedical Research Institute, Jeonbuk National University Hospital, Jeonju, South Korea.

**Keywords:** cancer, low-back pain, palliative care, radiofrequency, refractory pain

## Abstract

Cancer patients are increasing in number, with an increased lifespan and advances in cancer treatment. Palliative care physicians often encounter difficulties in caring for patients with pain. In addition to cancer-related pain, patients with cancer may suffer from various musculoskeletal diseases, resulting in significant functional limitations of physical activities of daily living. We present three cases illustrating methods to deal with nonspecific mechanical low-back pain in patients with advanced cancer. We provide our therapeutic experiences, focusing on the usefulness of radiofrequency treatment in palliative care in patients with cancer.

## Introduction

Although low-back pain (LBP) is known to be one of the most common health problems with a one-year prevalence estimated at 38%,^1^ the epidemiology of LBP in cancer patients has been poorly investigated and underestimated. LBP might be an important sign of more serious diseases in some cancer patients, such as spinal metastases. In this situation, the patients might present severe night pain or progressive and non-mechanical pain, which is considered a red flag for spinal metastasis.^2^

In contrast, the patients can also experience nonspecific mechanical LBP, which is not associated with cancer itself but with degenerative conditions of the spine. Nevertheless, the intensity of nonspecific LBP in cancer patients might be much more severe, leading to significant deterioration of functional abilities compared with noncancer patients.

Lumbar facet syndrome (LFS) is considered one of the most frequent degenerative spine diseases.^3^ LFS-related pain may present as LBP, which can be radiated to the buttock or leg according to the affected facet joints. The diagnosis of LFS is often challenging for pain physicians due to the low diagnostic accuracy of physical examination based on symptoms and signs and the unpredictability of radiological imaging, including magnetic resonance imaging (MRI) and computed tomography (CT).^4^

The degree of radiological facet joint abnormality detected by MRI is poorly correlated with LBP symptoms.^5^ Instead, lumbar medial branch block (MBB) is widely accepted as a useful diagnostic tool for LFS with moderate to strong strength of recommendation. Radiofrequency (RF) treatment of the medial branch as well as nerve block has been accepted as a successful therapeutic option for LFS.^4,6^

In this case series, we encountered three patients with different types of advanced-to-terminal cancers who were referred to our palliative care center for managing LBP. All patients were suspected of having LFS without evident spinal metastasis. We introduce our experience with successful treatment with medial branch RF for LBP in patients with cancer.

## Case Descriptions

Written informed consent for publication of this case series was obtained from each patient. We experienced three cases of successful RF treatment for nonspecific mechanical LBP in patients with cancer without definite spinal metastasis. The LBP-related clinical characteristics of the patients are presented in Table 1.

**Table 1.** Characteristics of the Low-Back Pain

	Case 1	Case 2	Case 3
Main complaint	LBP	LBP	LBP
Subjective description	Unexplainable severe LBP	Pain aggravation during standing or supine position He could not sleep in the supine position	Pain aggravation during supine position She had to sleep while sitting in a chair
NRS (0–10)	9	8	6
Physical examination	Direct tenderness on both L4/5 and L5/S1 facet	Direct tenderness on both L3/4 and L4/5 facet	Direct tenderness on right L5/S1 facet
Type of analgesics			
NSAIDs	Transdermal ketoprofen	None	None
Opioids, *oral* morphine equivalent dose (mg/day)	375	150	285
Adjuvants	None	None	None

LBP, low-back pain; NRS, numeric rating scale; NSAIDs, non-steroidal anti-inflammatory drugs.

### Case 1

A 46-year-old man visited our palliative pain center because of a recently aggravated LBP. The patient had received palliative chemotherapy for advanced gastric cancer with peritoneal metastasis for 19 months. However, radiological examinations such as CT revealed progressive disease, and end-of-life care was planned. The patient complained of severe and aching acute LBP with the worst intensity of 9 on the 11-point numeric rating scale (NRS). LBP was refractory even with a gradual increase in the dose of analgesics, including transdermal non-steroidal anti-inflammatory drugs (NSAIDs), transdermal fentanyl 100 μg/h, intravenous morphine 25 mg/day, and intranasal fentanyl spray 200 μg/day. The maximum opioid dose was equivalent to an oral morphine dose of 375 mg/day.

He had previously received celiac plexus neurolysis with 99% dehydrated alcohol due to cancer-related abdominal visceral pain six months before presentation, and the abdominal pain relieved. The patient was referred to our palliative care pain center. Although he could not describe his pain because of severe debility, he presented definite direct tenderness on both the L4/5 and L5/S1 lumbar facets on physical examination. The patient was suspected of having LFS, and we first considered MBB at L3, L4, and L5 bilaterally for diagnostic and therapeutic procedures.

The procedure was performed under fluoroscopic guidance after a previously accepted standard technique.^6^ Upon the procedure, the affected facet joints were rechecked by palpation using fluoroscopy. The correct final needle position, which was the junction of the superior articular and transverse processes, was confirmed using oblique, anteroposterior, and lateral fluoroscopic images. A total of 0.5 mL of 0.2% ropivacaine was injected at each site after confirming negative blood aspiration and appropriate contrast dye dispersion. After two repeated MBBs, the patient reported a 50% pain reduction (from 9 to 4 of the NRS).

### Case 2

A 70-year-old man visited our outpatient palliative care pain center because of acute LBP that had developed a month prior. The patient had previously received pylorus-preserving pancreaticoduodenectomy (PPPD) for pancreatic head cancer 24 months prior, and adjuvant concurrent chemoradiation therapy was administered. The LBP with an average NRS of 8/10 persisted despite taking oral opioids (equivalent to an oral morphine dose of 150 mg/day) and several pain interventions, such as trigger point injections. The patient's LBP negatively affected his quality of life. He had difficulty sleeping because he could not lie down in the supine position for several minutes.

He did not complain of abdominal discomfort even though the CT scan revealed cancer infiltration in the proximity of the superior mesenteric artery and multiple retroperitoneal lymph node metastases with peritoneal seeding. Physical examination revealed severe tenderness on palpation of the L3/4 and L4/5 lumbar facet joints. MBB at bilateral L2, L3, and L4 was performed, and the patient experienced pain reduction by 80% (from 8 to 4 of the NRS) after two repeated blocks.

### Case 3

A 65-year-old woman, diagnosed with common bile duct cancer with lung metastasis, was referred to our outpatient palliative care pain center because of LBP radiating to the right buttock area. She underwent PPPD and adjuvant chemotherapy for 34 months. CT findings revealed cancer infiltration in the mesenteric root and hematogenous lung metastasis. She reported that her pain had increased, especially in the supine position; therefore, she had to sleep while lying in a chair.

Although the patient was taking oral medications equivalent to an oral morphine dose of 285 mg/day, the pain persisted. After confirming severe tenderness on the right L5/S1 facet joint using fluoroscopy, diagnostic MBB at the right L4 and L5 was performed. After two repeated MBBs, she reported a pain reduction of 50% (from 6 to 3 of the worst pain NRS).

## RF Procedures

All patients were considered to have a positive response to diagnostic MBBs, and RF treatment was performed by an experienced pain physician. The RF procedures were performed using an RF generator (Cosman G4; Cosman Medical, Burlington, MA) using a thermocouple electrode needle. The target facet joints and segmental medial branches were visualized using fluoroscopy in the oblique view. A 22-gauge 10-cm RF needle with a 5-mm active tip (Abbott Medical, Plymouth, MN) was advanced adjacent to the medial branches.

**FIG. 1.**
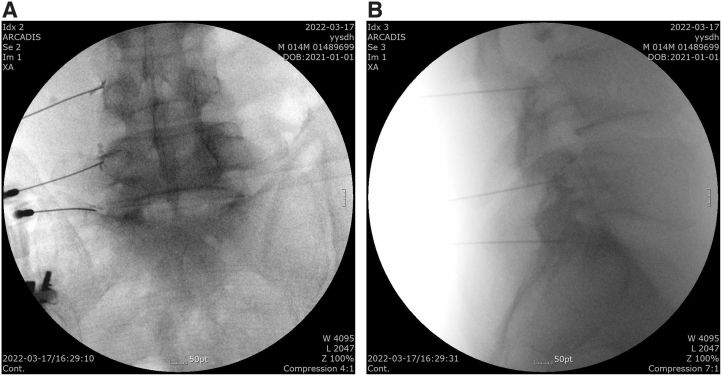
Anteroposterior **(A)** and lateral **(B)** radiography showing appropriate needle positioning with contrast dye during medial branch radiofrequency procedure.

Upon placement of the needle, the appropriate impedance was verified to be in the range of 300–600 Ω. Then, sensory stimulation was tested at 0.5–0.6 V with 50 Hz to ensure appropriate paresthesia and concordant pain. Motor stimulation at 1.0–1.2 V with 2 Hz was tested to detect muscle contraction in the multifidus muscle. The anteroposterior and lateral views of the appropriate RF needle placements to the medial branches with the dispersion of contrast dye are shown in Figure 1A and B in Case 1.

In Cases 1 and 3, pulsed RF treatment was delivered at 42°C and 45 V for 180 seconds, with a pulse frequency of 2 Hz and a pulse width of 20 ms for each site. In Case 2, a combined conventional and pulsed RF procedure was performed. Conventional RF was applied to the right L3 and left L4, where the most painful concordant LBP occurred. Before the lesioning, 0.5–1 mL of 2% lidocaine was injected for anesthesia.

The tip temperature was set at 80°C with a lesion time of 180 seconds at each location. At the rest of the target site, pulsed RF was performed using the same technique as in Cases 1 and 3. The procedural details for each patient are described in Table 2. At the four-week follow-up, all three patients reported remarkable and satisfactory pain relief. In addition, none of the patients reported complications after the procedure.

**Table 2.** Details of the Procedure

	Case 1	Case 2	Case 3
Diagnostic blocks	Medial branch block at bilateral L3, L4, and L5	Medial branch block at bilateral L2, L3, and L4	Medial branch block at right L4 and L5
Pain reduction (%) by diagnostic block	50%	80%	50%
Site of RF procedure	Pulsed RF at bilateral L3, L4, and L5 medial branch	- Conventional RF at right L2 and left L3 medial branch - Pulsed RF at right L3 and L4 and left L2 and L4 medial branch	Pulsed RF at right L4 and L5 medial branch
RF operation	42°C for 180 seconds for each site	- 80°C for 180 seconds for conventional RF - 42°C for 180 seconds for pulsed RF	42°C for 180 seconds for each site
Adverse reaction during RF procedure	None	None	None
Results at four weeks follow-up	Pain relief by 70%	Disappearance of LBP	Pain relief by 70%

RF, radiofrequency.

## Discussion

Many patients with cancer experience reduced physical activity in daily living during and after cancer treatment. The mechanism of the functional decline in cancer patients is complex and multifactorial and includes cancer-related factors, patients' comorbidities, psychosocial factors, and the consequences of anticancer treatment.^7^ Severe functional limitations of physical activities might be associated with various musculoskeletal dysfunctions, especially in advanced or end-stage cancer patients.^8,9^ Although the prevalence of musculoskeletal diseases among cancer patients is still unknown, various musculoskeletal diseases might occur due to the cancer burden itself or anticancer treatment.^10^

In the current cases, the cause of LBP seems to be LFS. LFS is a common degenerative spine disease that causes LBP, and its prevalence may increase with age.^11^ Lumbar facet joints represent a potential pain generator because the facet joint capsule and surrounding structures are richly imbued with nociceptors.^5^ With aging, the joints may undergo biomechanical changes such as capsular weakness and morphological changes of the articular surface. Degeneration, inflammation, and repetitive injury of the facet joints are the proposed pathophysiological mechanisms of LFS.^12^

There are some possible explanations for the development of LFS, particularly in patients with cancer. First, the acceleration of degenerative changes in the spine and the paraspinal muscles may compromise the proper biomechanics of the spine.^13,14^ Moreover, most cancer patients experience significant weight loss and resultant progressive loss of skeletal muscle mass (sarcopenia).^15^ Cooley et al reported that severe facet degeneration was associated with altered paraspinal muscle morphology.^14^

The authors think that sarcopenic changes in the paraspinal muscles might be one of the causes of LFS in these patients. Second, osteoporotic changes in the spine may be another cause of LBP. According to the study results of Gheita et al,^10^ mild-to-moderate osteoporotic changes of the spine are prevalent in cancer patients, especially in those with solid tumors, which might partly contribute to the deterioration of the biomechanics of the spine.

However, the diagnosis of LFS is challenging. There have been no reliable pathognomonic historical signs or physical examination findings, and radiological imaging is not routinely recommended because of the low diagnostic value for LFS.^5,6^ Instead, diagnostic blocks including intra-articular or MBB with a low-volume injection are the most accepted confirmative diagnostic methods.

In the current cases, radiological examinations such as MRI or CT scans were not available because the severe LBP made it difficult for them to lie down in the supine position for several minutes. Fortunately, they could bear the prone position during the diagnostic MBB. The positive response from low-volume MBB (<0.5 mL) helped to determine the cause of LBP in these patients. The role of diagnostic MBB is noteworthy, especially in severely debilitated patients, such as those with advanced cancer.

There are two types of RF treatment: conventional RF and pulsed RF. Conventional RF ablation of the lumbar medial branches is the traditional and standard RF technique for the treatment of LFS. The conventional RF current coagulates the target nerve with controlled heat and causes denervation of the painful facet joint. It proved the short- and long-term effectiveness of LFS treatment.^6,16^ Meanwhile, pulsed RF treatment uses short-pulsation low energy to minimize tissue injury.^17^

Although the exact therapeutic mechanisms of pulsed RF are unclear, temporal blockage of nerve signals, enhancement of the descending inhibitory pathway, and changes in gene expression have been suggested to be associated with short- and long-term therapeutic efficacy.^18–20^ Pulsed RF has expanded its role in various neuropathic and musculoskeletal pain interventions. In the current case, we successfully treated LBP in an advanced cancer patient with pulsed RF to the lumbar medial branches. The authors believe that these cases demonstrate that RF treatment may be a safe and useful therapeutic option for various painful diseases, especially for patients with cancer in palliative care.

## Abbreviations Used

CT: computed tomography
LBP: low-back pain
LFS: lumbar facet syndrome
MBB: medial branch block
MRI: magnetic resonance imaging
NRS: numeric rating scale
PPPD: pylorus-preserving pancreaticoduodenectomy
RF: radiofrequency
